# Martial arts practice and self-identity development among college students: evidence from an embodied psychological perspective

**DOI:** 10.3389/fpsyg.2026.1811149

**Published:** 2026-07-15

**Authors:** Jiabing Zhou, Cheng Zheng, Mengmeng Hu, Yali Liu

**Affiliations:** 1 Guangdong University of Petrochemical Technology, Maoming, Guangdong, China; 2 Nanjing Normal University, Nanjing, Jiangsu, China

**Keywords:** identity development, interoception, martial arts, psychological resilience, self-concept

## Abstract

**Background:**

Emerging adulthood (18–25 years) is a critical developmental period characterized by identity exploration and psychological vulnerability. College students frequently experience stress, identity confusion, and mental health challenges. Martial arts training has been associated with psychological benefits; however, its role in identity development and the mechanisms underlying this relationship remain unclear. Grounded in an embodied psychological framework, this study examined whether an 8-week structured martial arts intervention enhances self-identity development among college students and whether embodied awareness mediates this effect.

**Methods:**

An 8-week intervention study quasi-experimental pre-post design was employed with 58 undergraduate students (aged 18–25 years) recruited from Guangdong University of Petrochemical Technology, China. Participants were allocated to a Martial Arts Intervention Group (MAIG; *n* = 29) or a Waitlist Control Group (WCG; *n* = 29). The MAIG completed an 8-week instructor-led training program (3 sessions/week), while the WCG maintained usual activities. Self-identity (EIPQ), embodied awareness (MAIA-2), self-esteem (Rosenberg Scale), and resilience (CD-RISC-10) were assessed at baseline and post-intervention. Mixed-design ANOVA and bootstrapped mediation analyses (PROCESS Model 4; 5,000 resamples) were conducted.

**Results:**

Significant Time × Group interaction effects were observed for identity commitment, embodied awareness, self-esteem, and resilience (all *p* < 0.001). The intervention group showed large improvements across all outcomes (partial η^2^ = 0.618–0.781). Mediation analysis revealed that changes in embodied awareness significantly mediated the relationship between group assignment and changes in identity commitment (95% CI did not include zero), indicating partial mediation.

**Discussion:**

Findings support the embodied perspective, suggesting that structured martial arts training promotes identity consolidation through enhanced bodily awareness. Martial arts may serve as an effective, body-based intervention to foster psychological development and resilience among college students.

## 1 Introduction

The transition to college represents a critical developmental period during which students navigate complex identity formation processes while confronting unprecedented academic, social, and psychological challenges. Emerging adulthood, defined as the developmental period spanning approximately ages 18–25 (Arnett, 2000), continues to be recognized as a critical phase of identity exploration and psychological development (Arnett et al., 2014). Contemporary research indicates that approximately 31–35% of college students experience significant mental health concerns, including anxiety, depression, and identity confusion, which can substantially impair their academic performance and overall wellbeing (Auerbach et al., 2018). In this context, the exploration of interventions that simultaneously promote psychological resilience and facilitate self-identity development has emerged as a paramount concern in higher education research.

Martial arts practice has garnered increasing scholarly attention as a holistic developmental activity that transcends mere physical training, offering practitioners a structured framework for cultivating mental discipline, emotional regulation, and self-awareness (Moore et al., 2020; Twemlow et al., 2008). Unlike conventional forms of physical exercise, martial arts training encompasses philosophical dimensions, ritualistic practices, and hierarchical progression systems that collectively contribute to practitioners’ sense of self and personal growth (Fuller, 1988). Recent meta-analyses suggest that martial arts participation is associated with enhanced self-esteem, reduced aggression, and improved psychological wellbeing across diverse populations (Ciaccioni et al., 2024; Moore et al., 2020). Recent studies have further emphasized the role of martial arts training in fostering emotional reflexivity and psychological development through structured physical engagement (Maclean, 2021). Furthermore, research demonstrates that martial arts training enhances self-efficacy, individuals’ beliefs in their capabilities to successfully perform specific tasks, which serves as a fundamental mechanism for behavioral change and personal development (Bandura, 2000). In the present study, the term martial arts refer specifically to a structured beginner-level training program integrating selected technical and pedagogical elements from Karate and Taekwondo. The intervention emphasized coordinated movement, discipline, attentional control, embodied awareness, and regulated partner interaction rather than competitive combat or high-performance sport participation. Accordingly, the findings should be interpreted within the context of a structured educational martial arts program and not generalized to other forms of combat sports, mixed martial arts, or professional fighting disciplines, which may differ substantially in training objectives, intensity, and underlying philosophical foundations.

The embodied perspective provides a valuable theoretical framework for understanding how martial arts practice influence’s identity development. This approach posits that cognitive processes and self-concept are fundamentally grounded in bodily experiences and sensorimotor interactions with the environment (Varela et al., 2017). Through repeated physical practice, martial artists develop what scholars’ term “embodied knowledge,” a form of tacit understanding that integrates physical competence with cognitive and emotional dimensions (Wacquant, 2004). This embodied learning process may facilitate identity development by enabling students to experience themselves as competent, disciplined, and capable individuals through direct physical engagement. Moreover, the physical self-concept, defined as individuals’ perceptions of their physical abilities, appearance, strength, and conditioning, has been identified as a crucial component of overall self-esteem and psychological wellbeing (Fox and Corbin, 1989).

Despite growing interest in martial arts as a developmental intervention, empirical research examining the specific mechanisms through which martial arts practice influences college students’ self-identity remains limited. Previous studies have predominantly focused on children and adolescents (Lakes and Hoyt, 2004; Twemlow et al., 2008), leaving a significant gap in our understanding of how martial arts training affects emerging adults navigating the unique challenges of college life. While Arnett’s (2000) theory of emerging adulthood emphasizes identity exploration as a central developmental task during the college years, few studies have explicitly examined martial arts practice as a vehicle for identity formation in this population (Arnett, 2000). Furthermore, while existing research has documented associations between martial arts practice and various psychological outcomes, few studies have explicitly adopted an embodied psychological framework to elucidate the processes underlying these relationships.

The present study addresses these gaps by investigating the relationship between martial arts practice and self-identity development among college students through an embodied psychological lens.

Specifically, the present study examined whether participation in an 8-week structured martial arts intervention was associated with changes in identity commitment, embodied awareness, self-esteem, and psychological resilience among university students. Drawing upon embodied psychological theory, we further explored whether changes in embodied awareness were associated with changes in identity development following the intervention. Drawing on Bandura’s (2000) social cognitive theory and Fox and Corbin’s (1989) hierarchical model of physical self-perception, we hypothesize that sustained martial arts practice facilitates identity development through embodied experiences that promote self-efficacy, emotional regulation, and integrated self-awareness (Bandura, 2000; Fox and Corbin, 1989). By employing validated psychometric instruments and controlling for relevant demographic and psychological variables, this research aims to provide preliminary empirical evidence regarding the potential developmental benefits of structured martial arts participation among university students. Engagement in structured extracurricular activities has also been associated with enhanced wellbeing and a sense of belonging among university students (Winstone et al., 2022), further supporting the relevance of martial arts training as a developmental intervention in higher education contexts.

From an embodied perspective, martial arts training may uniquely facilitate identity development through multiple interacting mechanisms. First, repeated sensorimotor engagement and coordinated movement patterns promote embodied self-awareness, enabling individuals to experience the self as grounded in bodily action rather than abstract cognition. Second, structured progression systems (e.g., skill mastery, hierarchical advancement) provide continuous feedback that reinforces perceived competence and self-efficacy, both of which are central to identity consolidation. Third, ritualized practices emphasizing discipline, respect, and attentional control may support internalization of values and stable self-definition. Finally, controlled sparring and partner-based interaction expose individuals to manageable stress, fostering emotional regulation and resilience. Together, these embodied, cognitive, and social processes suggest that martial arts training may influence identity development through mechanisms that extend beyond conventional physical activity. In addition to identity-related constructs, self-esteem and psychological resilience were included as secondary outcomes due to their established roles in psychological adjustment and their frequent association with physical activity and martial arts training in prior research.

### 1.1 Research hypotheses

*H1:* Participants in the martial arts intervention group will demonstrate significantly greater increases in identity commitment compared to the control group.

*H2:* Martial arts training will lead to significant improvements in embodied awareness relative to the control group.

*H3:* It was hypothesized that changes in embodied awareness would be associated with, and potentially explain, part of the relationship between participation in the martial arts intervention and changes in identity commitment.

*H4:* Participants in the intervention group will show greater improvements in self-esteem and psychological resilience compared to controls.

## 2 Materials and methods

### 2.1 Participants and ethical considerations

A total of 58 undergraduate students (aged 18–25 years) were recruited through campus-wide advertisements and classroom announcements at Guangdong University of Petrochemical Technology, China. Participant recruitment was conducted between September and October 2025 through campus-wide advertisements, classroom announcements, and institutional communication channels. Interested students were screened for eligibility prior to enrolment. Consistent with the quasi-experimental design of the study, participants were allocated to either the Martial Arts Intervention Group (MAIG) or the Waitlist Control Group (WCG) based on their availability to attend the scheduled intervention sessions (Table 1). Students who were able to commit to the training schedule were assigned to the intervention group, whereas those who were unavailable during the scheduled training periods were assigned to the waitlist control group. This allocation approach was adopted to facilitate intervention implementation under real-world university conditions and should be considered when interpreting the findings. Inclusion criteria required participants to be full-time undergraduate students, medically cleared for moderate physical activity, and not engaged in formal martial arts training within the previous 12 months. Exclusion criteria included current psychiatric treatment, significant musculoskeletal injury, or participation in professional-level combat sports. Participants in the intervention group underwent structured training in disciplines including Karate and Taekwondo. The intervention was delivered by instructors holding nationally recognized martial arts coaching certifications in Karate and Taekwondo, with a minimum of 5 years of instructional experience in educational and community-based training settings. All instructors underwent orientation regarding study procedures to ensure consistency and fidelity of intervention delivery across sessions. All participants provided written informed consent prior to participation. Gender was not included as an analytical variable in the present study. Although previous research suggests that experiences in martial arts may differ by gender, the current study focused on general psychological outcomes. Future research is encouraged to examine gender-specific effects. The study received approval from the Institutional Ethics Committee of Guangdong University of Petrochemical Technology, China (Approval No.: IEC/GUPT/SPE821). The research was conducted in accordance with the ethical standards of the Declaration of Helsinki. Written informed consent was obtained from all participants prior to enrolment. All data were stored securely on password-protected institutional devices accessible only to the research team.

**TABLE 1:** Participant characteristics at baseline.

Variable	MAIG (*n* = 29) mean ± SD	WCG (*n* = 29) mean ± SD	Total (*N* = 58) mean ± SD
Age (years)	21.4 ± 2.0	21.1 ± 1.9	21.3 ± 1.9
Weight (kg)	60.8 ± 2.3	61.2 ± 2.5	61.0 ± 2.4
Height (cm)	168.2 ± 3.4	167.5 ± 3.6	167.9 ± 3.5
BMI (kg/m^2^)	21.50 ± 0.88	21.81 ± 0.94	21.66 ± 0.91

Baseline comparisons indicated no statistically significant differences between groups for demographic characteristics or primary outcome measures (all *p* > 0.05), suggesting reasonable comparability between the intervention and control groups prior to the intervention.

### 2.2 Study design

The present study employed an 8-week quasi-experimental pre-post intervention design to examine the association between participation in a structured martial arts training program and changes in self-identity development among college students. Assessments were conducted at two time points: baseline (T1) and post-intervention (T2). The study was conducted between October and December 2025 at Guangdong University of Petrochemical Technology, China.

### 2.3 Outcome measures

#### 2.3.1 Primary outcome measure

The primary outcome of the study was self-identity development, assessed using the Ego Identity Process Questionnaire (EIPQ) (Balistreri et al., 1995). The EIPQ is a 32-item validated instrument designed to measure two central dimensions of identity formation: identity exploration and identity commitment. Items are rated on a 6-point Likert scale ranging from 1 (strongly disagree) to 6 (strongly agree). Subscale scores were computed by summing the relevant items, with higher scores indicating stronger identity exploration and commitment. The exploration subscale assesses active questioning and consideration of alternatives, whereas the commitment subscale evaluates the degree of personal investment and stability in decision-making. Consistent with previous literature, internal consistency estimates typically range from α = 0.70–0.78 for exploration and α = 0.75–0.85 for commitment. In the present study, internal consistency was acceptable (Exploration α = 0.76; Commitment α = 0.80). Change in identity commitment from pre-intervention (T1) to post-intervention (T2) was considered the principal indicator of intervention effectiveness.

#### 2.3.2 Secondary outcome measures

Embodied awareness was assessed using the Multidimensional Assessment of Interoceptive Awareness (MAIA-2) (Mehling et al., 2018). The MAIA-2 consists of 37 items measuring multiple domains of interoceptive and body awareness, including attention regulation, emotional awareness, self-regulation, body listening, and trusting bodily sensations. Items are rated on a 6-point Likert scale. Total scores were calculated as the mean of all items, with higher scores reflecting greater embodied awareness. Published studies report internal consistency coefficients typically exceeding α = 0.85 for the total scale. In the present study, internal consistency was α = 0.87. Pre-post changes in embodied awareness were examined to evaluate whether the intervention enhanced body-based psychological processing.

Self-esteem was measured using the Rosenberg Self-Esteem Scale. This 10-item instrument assesses global self-worth using a 4-point Likert format ranging from strongly disagree to strongly agree (Rosenberg, 1965). Total scores were computed by summing items after reverse coding negatively worded statements, with higher scores indicating greater self-esteem. Previous research consistently reports internal consistency values between α = 0.80 and 0.90. In the present study, Cronbach’s alpha was α = 0.88. Pre-post changes in self-esteem were analyzed to determine whether martial arts training influenced global self-evaluative processes.

Psychological resilience was assessed using the Connor-Davidson Resilience Scale (10-item version) (Connor and Davidson, 2003). The CD-RISC-10 evaluates adaptability, stress tolerance, and persistence under adversity. Items are rated on a 5-point Likert scale ranging from 0 (not true at all) to 4 (true nearly all the time). Total scores were computed by summing items, with higher scores indicating greater resilience. Published reliability estimates typically range from α = 0.85 to 0.90. In the present study, internal consistency was α = 0.86. Changes in resilience were examined to assess adaptive psychological functioning following the intervention. Validated Chinese versions of all instruments were used in the present study. Specifically, the Chinese version of the Rosenberg Self-Esteem Scale was adapted and validated by Jiang et al. (2023), the Chinese version of the Connor-Davidson Resilience Scale by Yu and Zhang (2007), and the Chinese version of the MAIA-2 by Teng et al. (2022), all of which have showed acceptable reliability and validity in Chinese populations. These versions were developed through standardized translation and back-translation procedures and have showed acceptable psychometric properties in Chinese populations. No additional translation was performed in the present study. Previous studies validating the Chinese versions of these instruments have reported satisfactory psychometric properties, including good internal consistency (Cronbach’s α > 0.70) and construct validity. In the present study, internal consistency coefficients ranged from α = 0.76 to 0.88, further supporting the reliability of the measures.

### 2.4 Procedure

Baseline assessments (T1) were conducted during the first week of October 2025 prior to the initiation of the intervention. Participants completed all questionnaires under supervised classroom conditions to ensure standardized administration. The 8-week training intervention was conducted from October to December 2025. Post-intervention assessments (T2) were completed within 1 week following the final training session in December 2025. Participants were informed that participation was voluntary and that they could withdraw at any time without penalty. All responses were anonymized using coded identifiers prior to analysis.

### 2.5 Intervention protocol

The martial arts intervention was designed as a structured, instructor-led 8-week training program aimed at enhancing embodied awareness, psychological regulation, and identity-related processes. The program was standardized across all sessions to ensure intervention fidelity and reproducibility. Training content incorporated fundamental techniques derived from Karate and Taekwondo, emphasizing coordinated movement, controlled partner interaction, and reflective bodily awareness. Sessions were conducted three times per week and supervised by certified instructors with a minimum of 5 years of teaching experience. A detailed overview of the intervention structure is presented in Table 2. The intervention was not designed to provide discipline-specific mastery in either Karate or Taekwondo. Instead, selected beginner-level elements from both disciplines were integrated to create a standardized martial arts-based training program focused on coordinated movement, attentional control, self-regulation, discipline, and embodied awareness. This approach was adopted to provide participants with a broad range of structured movement experiences while maintaining consistency in instructional delivery across the intervention period. Because the primary aim of the study was to examine psychological and developmental outcomes rather than sport-specific technical proficiency, the program emphasized general martial arts principles and movement practices that could be delivered safely and consistently to novice participants. Consequently, the intervention should be interpreted as a martial arts-based developmental program rather than formal instruction within a single martial arts tradition (Jennings et al., 2022; Jennings, 2019).

**TABLE 2:** Structured 8-week martial arts training protocol.

Component	Description	Duration per session	Frequency	Intensity	Rationale
Program duration	8-Week structured intervention	–	3 Sessions/week	–	Consistent with behavioral intervention duration in psychological training studies
Session length	Total training time per session	60 Min	–	Moderate-to-vigorous	Standard duration used in martial arts-based interventions
Warm-up phase	Dynamic mobility drills, joint activation, light aerobic movement	10 Min	Each session	Low-to-moderate intensity	Injury prevention and physiological preparation
Technical skill training	Fundamental stances, strikes, blocks, footwork, coordinated movement patterns (Karate and Taekwondo basics)	25 Min	Each session	Moderate intensity	Motor learning, discipline, embodied movement awareness
Partner drills/controlled sparring	Structured partner practice focusing on timing, control, and regulated contact	15 Min	Each session	Moderate-to-vigorous intensity	Social interaction, emotional regulation, self-control
Cool-down and mindful breathing	Static stretching, guided breathing, body scanning exercises	10 Min	Each session	Low intensity	Enhancement of interoceptive awareness and embodied reflection

Exercise intensity classifications presented in Table 2 were based on the expected physical demands of each training component and instructor observation rather than direct physiological monitoring. Heart rate was not objectively measured during the intervention period; therefore, intensity categories should be interpreted as descriptive estimates of training demands.

Participants assigned to the waitlist control group did not receive any structured martial arts training during the 8-week study period (October–December 2025). They were instructed to maintain their usual academic routines and refrain from initiating any new organized physical training programs, particularly combat sports or martial arts activities. No behavioral or psychological intervention was administered to the control group during this period. This approach was implemented to control for potential maturation effects and repeated-measure testing influences. Upon completion of the post-intervention assessment (T2), control participants were offered the opportunity to participate in the martial arts training program.

#### 2.5.1 Intervention adherence

The intervention consisted of 24 planned training sessions delivered over the 8-week study period (three sessions per week). Consistent with the predefined adherence criterion, participants were required to attend at least 80% of scheduled sessions (minimum of 20 sessions) to be included in the final analysis. Participant attendance was monitored throughout the intervention period. Consistent with the predefined protocol, participants were required to attend at least 80% of scheduled sessions to be included in the final analysis. Attendance records were maintained by instructors at each training session. Overall adherence rates were calculated to evaluate intervention fidelity and participant engagement.

### 2.6 Statistical analysis

Statistical analyses were performed using SPSS Version 23. Data were screened for missing values, normality (skewness and kurtosis), and outliers prior to analysis. Descriptive statistics (means and standard deviations) were calculated for all study variables at both time points. A mixed-design repeated-measures analysis of variance (ANOVA) was conducted with Time (T1 vs. T2) as the within-subject factor and Group (Intervention vs. Control) as the between-subject factor to evaluate changes in identity exploration, identity commitment, and embodied awareness. Significant Time × Group interaction effects were interpreted as evidence of intervention-related change. Effect sizes were reported using partial eta squared (η^2^*p*). To test the embodied psychological mechanism, change scores (Δ = T2–T1) were calculated and analyzed using bootstrapped mediation analysis (PROCESS Macro, Model 4; 5,000 resamples) to determine whether changes in embodied awareness mediated changes in identity commitment. Indirect effects were considered statistically significant if the 95% bias-corrected confidence interval did not include zero. Within the intervention group, linear regression analyses were conducted to examine whether training attendance predicted magnitude of change in identity commitment. Statistical significance was set at *p* < 0.05 (two-tailed), and 95% confidence intervals were reported. To reduce the likelihood of Type I error and enhance robustness, results were interpreted using a combination of *p*-values, effect sizes, and 95% confidence intervals. Bootstrapping procedures were additionally employed for mediation analysis.

## 3 Results

Table 3 presents the descriptive statistics for all study variables at baseline (T1) and post-intervention (T2) for the Martial Arts Intervention Group (MAIG) and the Waitlist Control Group (WCG). At baseline, the MAIG showed a mean identity commitment score of 84.28 (SD = 7.51), which increased to 91.97 (SD = 6.69) following the 8-week intervention. In contrast, the WCG showed minimal change, with scores increasing slightly from 80.93 (SD = 6.97) at T1 to 81.10 (SD = 6.72) at T2. For embodied awareness, the MAIG exhibited a substantial increase from 102.14 (SD = 7.10) at baseline to 109.14 (SD = 7.19) post-intervention. The WCG remained relatively stable across time, with mean scores of 103.86 (SD = 9.09) at T1 and 103.93 (SD = 8.56) at T2. Regarding self-esteem, participants in the MAIG showed a notable improvement from 20.45 (SD = 2.38) to 24.72 (SD = 2.30) following the intervention. In comparison, the WCG showed only a marginal increase from 21.62 (SD = 2.31) at baseline to 21.93 (SD = 2.76) at post-test. Similarly, psychological resilience increased markedly in the MAIG, rising from 24.86 (SD = 3.53) at T1 to 29.69 (SD = 3.54) at T2. The WCG showed minimal change over the same period, with scores moving slightly from 26.24 (SD = 3.30) to 26.72 (SD = 3.38). Overall, descriptive trends indicate consistent pre-post improvements across all psychological variables in the MAIG, whereas the WCG showed negligible changes across the 8-week study period.

**TABLE 3:** Descriptive statistics for study variables at pre- and post-intervention.

Variable	Group	Pre-test mean (SD)	Post-test mean (SD)
Identity commitment	MAIG	84.28 (7.51)	91.97 (6.69)
	WCG	80.93 (6.97)	81.10 (6.72)
Embodied awareness	MAIG	102.14 (7.10)	109.14 (7.19)
	WCG	103.86 (9.09)	103.93 (8.56)
Self-esteem	MAIG	20.45 (2.38)	24.72 (2.30)
	WCG	21.62 (2.31)	21.93 (2.76)
Psychological resilience	MAIG	24.86 (3.53)	29.69 (3.54)
	WCG	26.24 (3.30)	26.72 (3.38)

Table 4 presents the results of the mixed-design ANOVA was conducted to examine Time (pre vs. post) × Group (MAIG vs. WCG) effects across psychological outcomes. Specifically, participants in the intervention group showed substantial improvements across all psychological variables, whereas the control group showed minimal changes over time. A mixed-design ANOVA revealed significant Time × Group interaction effects for all variables. Given the presence of significant interaction effects, the main effects of Time and Group were not interpreted independently, as the interaction indicates that changes over time differed between groups. For identity commitment, the interaction effect was statistically significant, *F*(1, 56) = 199.20, *p* < 0.001, partial η^2^ = 0.781. Similarly, embodied awareness showed a significant interaction effect, *F*(1, 56) = 163.15, *p* < 0.001, partial η^2^ = 0.728. Significant interaction effects were also observed for self-esteem, *F*(1, 56) = 132.99, *p* < 0.001, partial η^2^ = 0.704, and psychological resilience, *F*(1, 56) = 89.15, *p* < 0.001, partial η^2^ = 0.618. Full repeated-measures ANOVA results, including main effects and interaction effects, are presented in Supplementary Table S1.

**TABLE 4:** Time × group interaction effects for psychological outcomes.

Variable	Time × group interaction *F*(1, 56)	*P*-value	Partial η^2^
Identity commitment	199.20	<0.001	0.781
Embodied awareness	163.15	<0.001	0.728
Self-esteem	132.99	<0.001	0.704
Psychological resilience	89.15	<0.001	0.618

**FIGURE 1:**
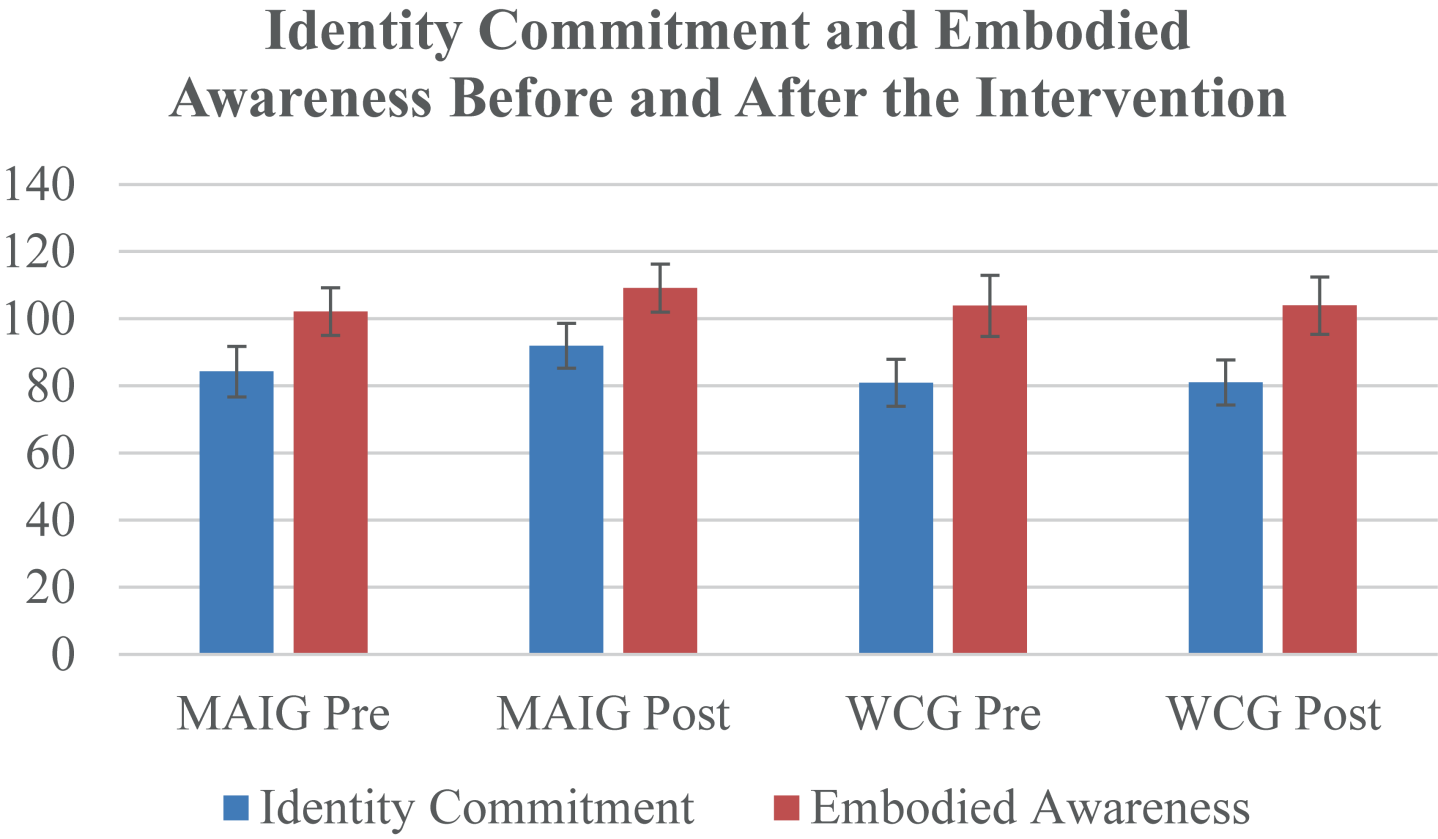
Pre- and post-intervention changes in identity commitment and embodied awareness among the Martial Arts Intervention Group (MAIG) and Waitlist Control Group (WCG). Values are presented as mean ± standard deviation.

**FIGURE 2:**
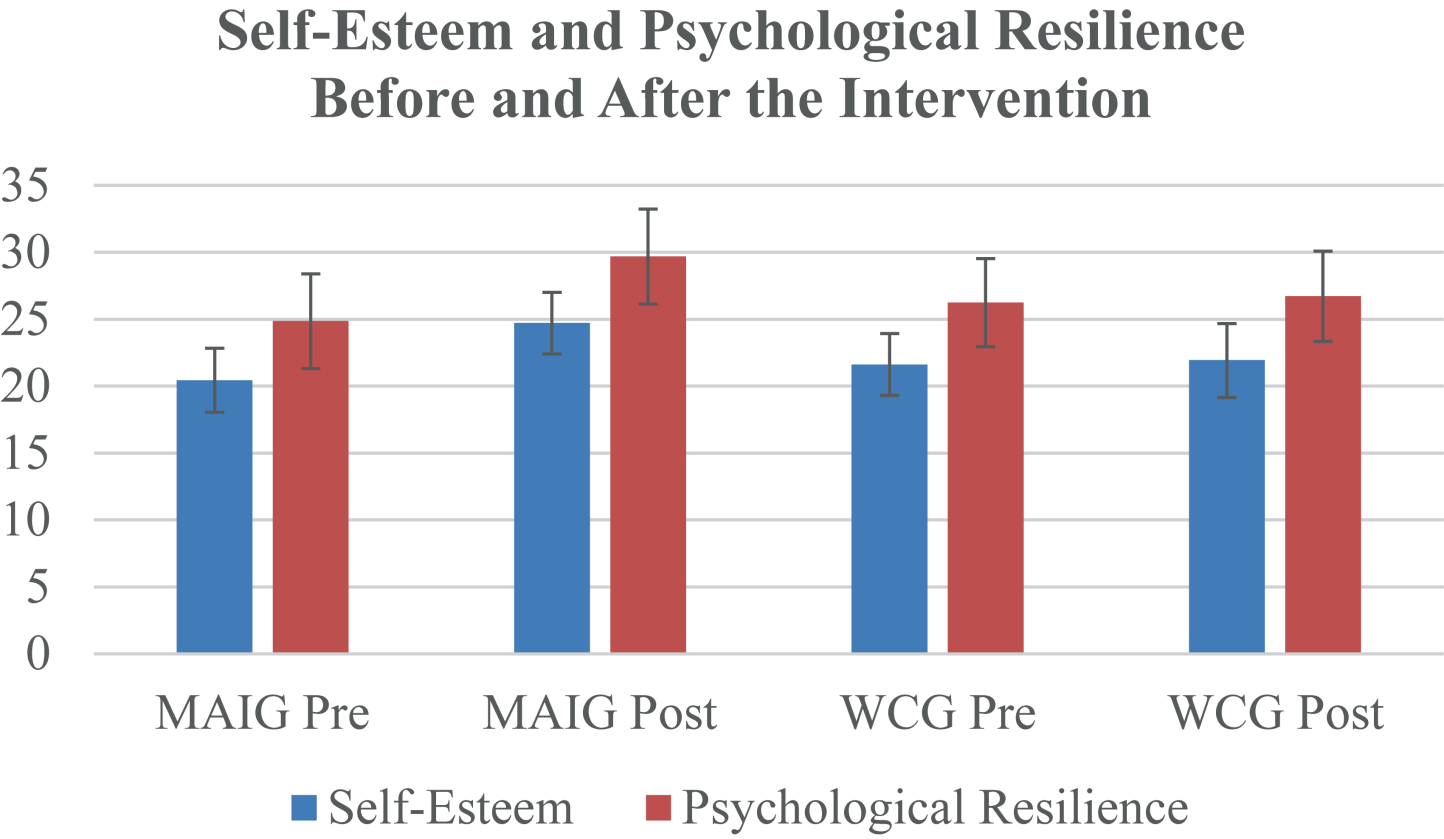
Pre- and post-intervention changes in self-esteem and psychological resilience among the Martial Arts Intervention Group (MAIG) and Waitlist Control Group (WCG). Values are presented as mean ± standard deviation.

For visual presentation of intervention-related changes, Figure 1 illustrates pre- and post-intervention changes in identity commitment and embodied awareness, whereas Figure 2 presents changes in self-esteem and psychological resilience. As illustrated in Figures 1, 2, participants in the Martial Arts Intervention Group (MAIG) showed greater pre-post improvements across all psychological outcomes than those in the Waitlist Control Group (WCG), where changes were comparatively minimal.

To further examine the significant interaction effects, Bonferroni-adjusted pairwise comparisons were conducted (see Table 5). Results showed that participants in the intervention group showed significant improvements from pre- to post-intervention across all variables, including identity commitment, embodied awareness, self-esteem, and psychological resilience (all *p* < 0.001). In contrast, no statistically significant changes were observed in the control group after Bonferroni correction (all *p* > 0.05). These findings indicate that the interaction effects were primarily driven by improvements in the intervention group.

**TABLE 5:** Bonferroni-adjusted pairwise comparisons (Pre vs. Post within each group).

Variable	Group	*T*-value	*P*-value	*p* (Bonferroni adjusted)
Identity commitment	Experimental	−17.76	<0.001	<0.001
	Control	−0.56	0.583	1.000
Embodied awareness	Experimental	−18.85	<0.001	<0.001
	Control	−0.16	0.873	1.000
Self-esteem	Experimental	−18.01	<0.001	<0.001
	Control	−1.25	0.222	0.889
Psychological resilience	Experimental	−12.99	<0.001	<0.001
	Control	−1.82	0.080	0.319

Independent samples *t*-tests were conducted to compare pre-post change scores (Δ = T2 − T1) between the Martial Arts Intervention Group (MAIG) and the Waitlist Control Group (WCG). Results revealed statistically significant differences across all psychological variables. For identity commitment, the MAIG showed a substantially greater increase (*M* = 7.69, SD = 2.05) compared to the WCG (*M* = 0.17, SD = 1.81), *t* = 14.11, *p* < 0.001. Similarly, embodied awareness increased significantly more in the MAIG (*M* = 7.00, SD = 2.47) than in the WCG (*M* = 0.07, SD = 2.14), *t* = 12.25, *p* < 0.001. Significant group differences were also observed for self-esteem, with the MAIG showing a mean increase of 4.28 (SD = 1.56) compared to 0.31 (SD = 1.42) in the WCG, *t* = 11.53, *p* < 0.001. Likewise, psychological resilience increased more prominently in the MAIG (*M* = 4.83, SD = 2.15) relative to the WCG (*M* = 0.48, SD = 1.89), *t* = 9.51, *p* < 0.001. Overall, the intervention group showed significantly greater improvements across all measured psychological outcomes compared to the control group, indicating strong intervention-related effects over the 8-week period (Table 6).

**TABLE 6:** Group differences in change scores (Δ) following the 8-week intervention.

Variable	Experimental mean change (SD)	Control mean change (SD)	*t*	*P*-value
Identity commitment (Δ)	7.69 (2.05)	0.17 (1.81)	14.11	<0.001
Embodied awareness (Δ)	7.00 (2.47)	0.07 (2.14)	12.25	<0.001
Self-esteem (Δ)	4.28 (1.56)	0.31 (1.42)	11.53	<0.001
Psychological resilience (Δ)	4.83 (2.15)	0.48 (1.89)	9.51	<0.001

Effect size analyses indicated that the intervention produced extremely large effects across all psychological outcomes. For identity commitment, the between-group difference in change scores yielded a Cohen’s d of 3.71, with a partial eta squared (η^2^*p*) of 0.78, indicating that approximately 78% of the variance in change scores was attributable to the intervention effect. The mean difference between groups was 7.52, with a 95% confidence interval ranging from 6.47 to 8.56, suggesting a robust and precise intervention effect. Similarly, embodied awareness showed a very large effect size (Cohen’s *d* = 3.22) and a partial eta squared of 0.72. The mean difference between groups was 6.93, with a 95% confidence interval of 5.82–8.04, further confirming substantial intervention-related improvement. For self-esteem, the effect size was also large (Cohen’s *d* = 3.03; η^2^*p* = 0.70), with a mean difference of 3.97 and a 95% confidence interval between 3.29 and 4.64. Psychological resilience showed a large effect (Cohen’s *d* = 2.50; η^2^*p* = 0.61), with a mean difference of 4.34 and a 95% confidence interval from 3.45 to 5.24. Overall, all effect sizes exceeded conventional benchmarks for large effects, and none of the confidence intervals included zero, indicating statistically and practically significant improvements in the intervention group relative to the control group (Table 7).

**TABLE 7:** Effect sizes and confidence intervals for between-group differences.

Variable	Cohen’s d	Partial η^2^ (η^2^p)	Mean difference	95% CI (lower)	95% CI (upper)
Identity commitment (Δ)	3.71	0.78	7.52	6.47	8.56
Embodied awareness (Δ)	3.22	0.72	6.93	5.82	8.04
Self-esteem (Δ)	3.03	0.70	3.97	3.29	4.64
Psychological resilience (Δ)	2.50	0.61	4.34	3.45	5.24

Table 8 examine the embodied psychological mechanism underlying the intervention effects, a bootstrapped mediation analysis was conducted using PROCESS Model 4 with 5,000 resamples. Change in embodied awareness (ΔEA) was specified as the mediator, and change in identity commitment (ΔIC) was specified as the outcome variable. Results indicated a statistically significant indirect effect of group assignment on change in identity commitment through change in embodied awareness (Indirect Effect = 5.65). The 95% bias-corrected bootstrapped confidence interval ranged from 4.46 to 6.79, and because this interval did not include zero, the mediation effect was considered statistically significant. The direct effect of group assignment on change in identity commitment remained significant after accounting for the mediator, indicating partial mediation. The total effect of the intervention on identity commitment was also statistically significant, confirming that participation in the 8-week martial arts program substantially contributed to improvements in identity commitment both directly and indirectly through enhanced embodied awareness. These findings support the hypothesized embodied psychological framework, suggesting that increases in embodied awareness represent a key mechanism through which martial arts training promotes identity development.

**TABLE 8:** Mediation analysis examining the indirect effect of embodied awareness on identity commitment.

Effect type	Coefficient	95% Boot CI (lower)	95% Boot CI (upper)	Significant
Indirect effect (group →ΔEA →ΔIC)	5.65	4.46	6.79	Yes
Direct effect (group →ΔIC)	Significant	–	–	Yes
Total effect	Significant	–	–	Yes

Mediator: Change in Embodied Awareness (ΔEA), Outcome: Change in Identity Commitment (ΔIC).

## 4 Discussion

The present pre-post design-controlled study investigated whether an 8-week structured martial arts intervention was associated with improvements in self-identity development among college students and whether embodied awareness mediates this effect. The findings suggested that participants who completed the structured martial arts intervention exhibited greater improvements in identity commitment, embodied awareness, self-esteem, and psychological resilience than participants in the waitlist control group. Although causal interpretations should be made cautiously due to the quasi-experimental design, the pattern of results suggests that participation in the intervention was associated with meaningful psychological changes over the 8-week period. Mediation analysis further revealed that change in embodied awareness significantly mediated the relationship between intervention participation and identity commitment. These findings support an embodied psychological framework of identity development and extend prior research on martial arts-based interventions. The findings of the present study provide clear support for the proposed hypotheses. Overall, the observed findings were generally consistent with the proposed hypotheses. Participants in the intervention group demonstrated greater improvements in identity commitment, embodied awareness, self-esteem, and psychological resilience than those in the waitlist control group. In addition, changes in embodied awareness were statistically associated with changes in identity commitment, consistent with the proposed embodied psychological perspective. However, given the quasi-experimental design, non-randomized group allocation, and reliance on self-report measures, these findings should be interpreted cautiously and should not be considered definitive evidence of causal relationships.

The observed increase in identity commitment in the intervention group aligns with developmental models of identity formation proposed by Kemph (1969) and operationalized in contemporary identity process models (Balistreri et al., 1995; Kemph, 1969). Identity commitment reflects consolidation of self-definition and personal investment in values and goals. Structured martial arts training provides repeated mastery experiences, progressive skill acquisition, and value-based instruction (discipline, respect, perseverance), all of which may facilitate identity consolidation.

Previous intervention studies have shown that martial arts participation enhances psychosocial development and self-concept among youth and adolescents. Lakes and Hoyt (2004) demonstrated that a school-based Taekwondo program significantly improved self-regulation and prosocial behavior compared to physical education controls (Lakes and Hoyt, 2004). Similarly, Moore et al. (2021) reported that a martial arts-based resilience program improved psychological resilience and emotional regulation in adolescents (Moore et al., 2021). These findings are consistent with recent theoretical perspectives suggesting that martial arts contribute to psychological wellbeing through embodied, social, and culturally structured practices (Tadesse, 2017). Although these studies focused primarily on resilience and self-regulation, the present findings extend this work by demonstrating direct improvements in identity commitment, a core developmental construct in emerging adulthood.

### 4.1 Mechanisms underlying martial arts–based identity development

The present findings can be more deeply understood through an embodied framework that explains not only whether martial arts training is effective, but why it facilitates identity development. One key mechanism is embodied self-awareness, whereby repeated motor engagement and interoceptive attention enhance the integration of bodily and cognitive aspects of the self. This aligns with embodied cognition theory, which posits that higher-order psychological processes are grounded in sensorimotor experience. Additionally, martial arts training provides structured mastery experiences that strengthen self-efficacy, a critical component of identity commitment. The hierarchical and ritualized nature of martial arts practice may further contribute by reinforcing internalization of values such as discipline and perseverance. Finally, controlled sparring and partner interaction create opportunities for emotional regulation under manageable stress conditions, thereby enhancing resilience and psychological stability. Together, these mechanisms suggest that martial arts training operates through an integrated system of embodied, cognitive, and social processes that promote identity consolidation.

A central contribution of this study is the demonstration that embodied awareness mediates the relationship between martial arts training and identity development. Embodied cognition theory posits that higher-order psychological processes are grounded in bodily states and sensorimotor experience (Wilson, 2002). Interoceptive awareness, the perception and interpretation of internal bodily signals, has been linked to emotional regulation, self-coherence, and adaptive functioning (Mehling et al., 2012).

The MAIA-2 has demonstrated strong psychometric validity in assessing multidimensional interoceptive awareness (Mehling et al., 2018). Enhanced interoceptive awareness has been associated with improved emotional stability and reduced psychological distress (Füstös et al., 2013). The present findings suggest that martial arts training, through controlled breathing, posture regulation, movement precision, and attentional focus, strengthens embodied awareness, which in turn promotes identity commitment. This mechanism is theoretically coherent. Identity commitment involves clarity and stability in self-definition; increased bodily awareness may reduce internal ambiguity and enhance integration of emotional and cognitive self-representations. Thus, embodied awareness may serve as a bridge between physical training and psychological consolidation.

The intervention group also showed substantial gains in self-esteem and psychological resilience. Martial arts training provides structured mastery experiences, which are foundational to self-efficacy and self-worth development (Bandura, 2000). Meta-analytic research indicates that physical activity interventions generally produce small-to-moderate improvements in self-esteem (Spence et al., 2005), but martial arts programs often yield stronger effects due to their emphasis on discipline, progression, and symbolic achievement.

Regarding resilience, Connor and Davidson (2003) conceptualized resilience as adaptive coping under stress. Controlled martial arts sparring and progressively challenging drills may simulate stress exposure in a structured and supportive environment, fostering adaptive coping skills. Recent randomized controlled trials have demonstrated that martial arts interventions improve resilience and psychological functioning in youth populations (Moore et al., 2021). The present findings replicate and extend these effects to emerging adult university students.

The partial eta squared values (0.618–0.781) observed in this study indicate large intervention effects according to conventional benchmarks (Cohen, 2013). Such effect sizes are uncommon in psychosocial intervention research and suggest that the embodied, skill-based, and socially structured nature of martial arts training may produce synergistic psychological benefits beyond conventional exercise programs.

This study integrates identity development theory with embodied cognition and physical activity research. While identity development has traditionally been examined through cognitive and social lenses, the present findings suggest that body-based interventions may directly influence identity consolidation processes. This supports contemporary embodied self-models, which argue that self-representation is grounded in sensorimotor and interoceptive processes (Damasio, 1999). By demonstrating mediation through embodied awareness, this study provides empirical support for a mechanism-based model of martial arts-induced psychological development. Although the findings were encouraging, several limitations should be acknowledged. Because group allocation was based on participant availability rather than random assignment, the possibility of selection bias cannot be entirely excluded. Consequently, causal interpretations regarding the observed intervention-related changes should be made with caution. Additionally, gender was not analyzed in the present study, which may influence how individuals experience martial arts training. Future studies should explore potential gender differences.

Several limitations should be acknowledged when interpreting the findings. First, the study employed a quasi-experimental design with a passive waitlist control group, limiting the extent to which causal conclusions can be drawn regarding the specific effects of martial arts training. Second, participants’ prior physical activity and sport participation histories were not systematically assessed and may have influenced psychological outcomes. Third, all outcome measures were self-reported, introducing the possibility of response bias and shared method variance. Fourth, the absence of an active comparison group prevents differentiation between martial arts-specific effects and broader benefits associated with structured physical activity, social interaction, or participant engagement. Finally, no follow-up assessments were conducted; therefore, the long-term sustainability of the observed changes remains unknown.

## 5 Conclusion

The present study suggests that participation in an 8-week structured martial arts-based training program was associated with improvements in identity commitment, embodied awareness, self-esteem, and psychological resilience among university students. Compared with participants in the waitlist control group, those who completed the intervention showed greater positive changes across all measured psychological outcomes. Furthermore, changes in embodied awareness were statistically associated with changes in identity commitment, consistent with the proposed embodied psychological perspective. These findings contribute preliminary evidence supporting the potential value of martial arts-based interventions for promoting psychological development during emerging adulthood. However, the results should be interpreted with caution given the quasi-experimental design, non-randomized group allocation based on participant availability, reliance on self-report measures, absence of an active comparison group, and lack of long-term follow-up assessment. Consequently, it cannot be determined whether the observed improvements were attributable specifically to martial arts training or to broader factors associated with structured physical activity, social engagement, or participation in a supervised intervention. Future research should employ randomized controlled designs, include active comparison conditions, incorporate objective behavioral and physiological indicators, and examine the long-term sustainability of intervention-related changes. Despite these limitations, the findings indicate that structured martial arts-based training may represent a promising approach for supporting identity-related and psychosocial development among university students.

## Data Availability

The original contributions presented in this study are included in this article/Supplementary material, further inquiries can be directed to the corresponding author.
