# Supplementary material for: Martial arts practice and self-identity development among college students: evidence from an embodied psychological perspective
Source: Front Psychol. 2026 Jul 15;17:1811149. doi: 10.3389/fpsyg.2026.1811149 (PMC13414969; doi:10.3389/fpsyg.2026.1811149)
Supplement: Supplementary file 2 [file Table_2.docx]

**Supplementary Table S1. Full Repeated-Measures ANOVA Results**

| **Variable** | **Effect** | **F(df)** | **p** | **Partial η²** |
| --- | --- | --- | --- | --- |
| **Identity Commitment** | Time | F (1,56) = 217.90 | < .001 | .796 |
|  | Group | F (1,56) = 15.34 | < .001 | .215 |
|  | Time × Group | F (1,56) = 199.20 | < .001 | .781 |
| **Embodied Awareness** | Time | F (1,56) = 156.14 | < .001 | .736 |
|  | Group | F (1,56) = 0.69 | .409 | .012 |
|  | Time × Group | F (1,56) = 150.11 | < .001 | .728 |
| **Self-Esteem** | Time | F (1,56) = 177.90 | < .001 | .761 |
|  | Group | F (1,56) = 1.71 | .196 | .030 |
|  | Time × Group | F (1,56) = 132.99 | < .001 | .704 |
| **Psychological Resilience** | Time | F (1,56) = 135.18 | < .001 | .707 |
|  | Group | F (1,56) = 0.82 | .368 | .014 |
|  | Time × Group | F (1,56) = 89.15 | < .001 | .618 |

Note: Time represents within-subject effects (pre vs. post), Group represents between-subject effects (intervention vs. control), and Time × Group represents interaction effects.
